# Psychopathic traits influence amygdala–anterior cingulate cortex connectivity during facial emotion processing

**DOI:** 10.1093/scan/nsy019

**Published:** 2018-04-12

**Authors:** Michael P Ewbank, Luca Passamonti, Cindy C Hagan, Ian M Goodyer, Andrew J Calder, Graeme Fairchild

**Affiliations:** 1 Medical Research Council Cognition and Brain Sciences Unit, University of Cambridge, Cambridge, UK; 2 Department of Clinical Neurosciences, University of Cambridge, Cambridge, UK; 3 Department of Psychiatry, University of Cambridge, Cambridge, UK; 4 Division of Humanities and Social Sciences, California Institute of Technology, Pasadena, CA, USA; 5 Department of Psychology, University of Bath, Bath, UK

**Keywords:** conduct disorder, psychopathy, callous–unemotional traits, connectivity, fMRI, emotion

## Abstract

There is accumulating evidence that youths with antisocial behavior or psychopathic traits show deficits in facial emotion recognition, but little is known about the neural mechanisms underlying these impairments. A number of neuroimaging studies have investigated brain activity during facial emotion processing in youths with Conduct Disorder (CD) and adults with psychopathy, but few of these studies tested for group differences in effective connectivity—i.e. changes in connectivity during emotion processing. Using functional magnetic resonance imaging and psycho-physiological interaction methods, we investigated the impact of CD and psychopathic traits on amygdala activity and effective connectivity in 46 male youths with CD and 25 typically-developing controls when processing emotional faces. All participants were aged 16–21 years. Relative to controls, youths with CD showed reduced amygdala activity when processing angry or sad faces relative to neutral faces, but the groups did not significantly differ in amygdala-related effective connectivity. In contrast, psychopathic traits were negatively correlated with amygdala–ventral anterior cingulate cortex connectivity for angry *vs* neutral faces, but were unrelated to amygdala responses to angry or sad faces. These findings suggest that CD and psychopathic traits have differential effects on amygdala activation and functional interactions between limbic regions during facial emotion processing.

## Introduction

There are now a large number of studies showing impairments in facial emotion recognition in antisocial populations, with studies of children, adolescents and adults all providing evidence that antisocial behavior and psychopathic traits are associated with emotion recognition difficulties (Dolan and Fullam, 2006; Marsh and Blair, 2008; Fairchild *et al.*, 2009; Fairchild *et al.*, 2010; Dawel *et al.*, 2012). However, despite the consistency of these findings and their potential clinical significance, relatively little is known about the neural mechanisms underlying these deficits. Dysfunction of the amygdala, anterior cingulate cortex (ACC), anterior insula and ventromedial prefrontal cortex (vmPFC)/orbitofrontal cortex (OFC) are considered to be plausible candidates (Blair, 2013). In line with this hypothesis, neuroimaging studies have revealed altered amygdala, insula and OFC activity during facial emotion processing in adolescents with Conduct Disorder (CD) or Oppositional Defiant Disorder (ODD) (Marsh *et al.*, 2008; Passamonti *et al.*, 2010; Fairchild *et al.*, 2014), and children with conduct problems and elevated psychopathic traits (Jones *et al.*, 2009; Viding *et al.*, 2012). Atypical ACC deactivation during the processing of emotional images has also been reported in adolescents with CD (Sterzer *et al.*, 2005). However, it is still unclear whether functional interactions between these components of the limbic system are abnormal in youths with CD or psychopathic traits. Studying functional connectivity in these populations is important as this has the potential to shift our understanding of brain function from a modular view toward one of integrated circuits, as well as deepening our understanding of the brain mechanisms underlying CD and psychopathy. Connectivity analyses may also be informative in interpreting previous findings obtained in antisocial populations, such as differences in amygdala activity when viewing emotional faces, which could be driven by reduced or increased regulation of the amygdala by linked brain regions.

Despite these potential advantages of connectivity methods, relatively few studies have investigated functional connectivity between the amygdala and associated limbic regions involved in regulating emotional responses, such as the ACC or the OFC, in antisocial populations. Using a facial emotion processing task, Marsh *et al.* (2008) found a negative correlation between psychopathic traits and amygdala–vmPFC functional connectivity in a small group of adolescents with disruptive behavior disorders (DBDs; *n* = 9). Marsh *et al.* (2011) subsequently reported reduced amygdala–OFC functional connectivity in adolescents with DBDs and psychopathic traits, relative to controls, during a moral decision-making task. A more recent study used resting-state fMRI to characterize the intrinsic functional connectivity of the basolateral (BLA) and centromedial (CMA) nuclei of the amygdala in youths with CD and callous–unemotional (CU) traits, relative to youths with CD without CU traits, and age-matched healthy controls (HCs) (Aghajani *et al.*, 2017). Relative to the other groups, youths with CD and CU traits showed increased connectivity between the BLA and ventral ACC (vACC), but lower connectivity between the CMA and vmPFC.

However, the connectivity analysis method used in the studies by Marsh *et al.* only tests for correlations between the fMRI time series of the respective regions and, as such, could reflect group differences in baseline or intrinsic connectivity rather than being specifically related to connectivity during task performance. The latter study by Aghajani *et al.* (2017) supports this idea, by showing altered functional connectivity in youths with CD and CU traits under resting state conditions. In contrast, effective connectivity methods, such as psycho-physiological interaction (PPI) analyses, enable researchers to study how the connectivity between brain regions is modulated or altered by specific task demands or stimulus characteristics (e.g. processing angry compared to neutral faces) (O’Reilly *et al.*, 2012). Consequently, these methods have the potential to detect differences in effective connectivity specifically during emotion processing.

Coccaro *et al.* (2007) used PPI methods to study adults with intermittent explosive disorder (IED), a psychiatric condition characterized by impulsive aggression. Relative to HCs, participants with IED showed reduced amygdala–vmPFC effective connectivity, and increased amygdala responses, when viewing angry faces (Coccaro *et al.*, 2007). Thus, the higher amygdala responses observed during anger processing may have been driven by weaker top-down regulation of the amygdala in the IED group.

Another study investigated connectivity changes between the amygdala and the face processing network in adult psychopaths using a matching task involving emotional faces (Contreras-Rodriguez *et al.*, 2014). The psychopathic group showed reduced effective connectivity between the amygdala and the primary visual cortex, fusiform gyrus, parietal cortex, and dorsolateral prefrontal cortex, relative to controls. However, these connectivity changes were unrelated to psychopathy scores, so this effect may have been explained by group differences in aggressive behavior (as the psychopaths were all violent offenders), rather than psychopathic traits. Likewise, in the studies by Marsh *et al.* (2008, 2011), all of the youths with psychopathic traits also had DBDs, so group differences in functional connectivity may have been driven by psychopathic traits or antisocial behavior.

To our knowledge, this study is the first to use effective connectivity methods to investigate connectivity in young people with CD and examine whether there are dimensional relationships between CD symptoms or variations in overall psychopathic traits and effective connectivity. To achieve these aims, we recruited additional subjects to add to an existing sample of adolescents with CD. While our previous study with an overlapping sample showed reduced amygdala responses to angry relative to neutral faces in adolescents with CD compared to HCs (Passamonti *et al.*, 2010), connectivity between the amygdala and other brain regions was not investigated.

Given previous work using the same task showing that connectivity between the amygdala and vACC is less negative in healthy adults who are high in reward drive (Passamonti *et al.*, 2008), a personality trait related to externalizing behavior, and evidence of dense anatomical connections between the vACC and amygdala (Ghashghaei *et al.*, 2007), we predicted that negative changes in amygdala–vACC effective connectivity during the processing of emotional *vs* neutral faces would be attenuated in adolescents with CD relative to HCs. We also tested for group differences in effective connectivity between the amygdala and the rest of the brain using an unbiased, whole-brain approach. As previous theoretical and empirical work suggests that there may be qualitative or quantitative differences between childhood-onset and adolescence-onset forms of CD (Moffitt, 1993; Fairchild *et al.*, 2013), we investigated whether these subgroups differed in terms of effective connectivity.

We also assessed the impact of psychopathic traits and CU traits more specifically on effective connectivity. We predicted that the negative changes in amygdala-vACC connectivity typically observed in healthy individuals in response to emotional *vs* neutral faces would be attenuated (i.e. closer to 0) in those with elevated psychopathic or CU traits. Finally, as previous studies have reported significant correlations between CD symptoms and brain structure (Fairchild *et al.*, 2011) or activity (Passamonti *et al.*, 2010), we tested for correlations between CD symptoms and amygdala–vACC effective connectivity, predicting that negative changes in connectivity would be attenuated in participants with more, compared to those with fewer, CD symptoms.

## Materials and methods

### Participants

Seventy-one male adolescents and young adults with CD were recruited from schools, pupil referral units and the Cambridge Youth Offending Service. Exclusion criteria included: Intelligence Quotient (IQ) < 85, as estimated using the Wechsler Abbreviated Scale of Intelligence (WASI), the presence of a pervasive developmental disorder (e.g. autism) or chronic physical illness. An HC group (no history of CD/ODD and no current psychiatric illness) of 25 male adolescents were recruited from schools and colleges. All subjects were aged 16–21 years. To equate groups in terms of estimated IQ, controls with estimated full-scale IQs > 115 were excluded. Fourteen CD subjects were excluded due to excessive head movement (>2 mm) in the scanner, three due to signal dropout in the medial temporal/OFC regions and a further two due to poor behavioral performance (<60% accuracy). This left us with 46 CD and 25 HC participants with usable data (Table 1), 60 of whom overlapped with the sample described in Passamonti *et al.* (2010). The study was approved by the Suffolk National Health Service Research Ethics Committee and all participants gave written informed consent.

**Table 1.** Characteristics of the participants included in the functional magnetic resonance imaging analyses

Measure	CON (*n* = 25)		AO–CD (*n* = 22)		CO–CD (*n* = 24)		ANOVA	Post-hoc
	Mean	s.d.	Mean	s.d.	Mean	s.d.	*P* values	
Age (years)	18.3	0.9	17.6	1.1	18.1	1.3	0.09	
Estimated full-scale IQ	101.9	8.4	100.7	8.7	97.6	7.6	0.17	
Psychopathic traits (YPI total)	95	15	115	15	120	20	<0.001	CON < (AO, CO)
YPI callous-unemotional subscale	30	5	35	5	35	5	<0.001	CON < (AO, CO)
Lifetime CD symptoms	0.4	0.6	7	2.3	9.2	1.8	<0.001	CON < AO < CO
Current CD symptoms	0	0.2	4.3	1.8	4.9	2.4	<0.001	CON < (AO, CO)
	*N*	**%**	*N*	**%**	*N*	**%**	*P*	
ACORN socioeconomic status*:								
Wealthy achievers (1)	5	18.5	2	7.4	0	0	0.02	CON > CO
Urban prosperity (2)	7	25.9	6	22.2	2	5.6		
Comfortably off (3)	6	22.2	6	22.2	16	44.4		
Moderate means (4)	2	7.4	1	3.7	4	11.1		
Hard-pressed (5)	7	25.9	12	44.4	14	38.9		
*Ethnicity:*								
Caucasian	26	96.3	25	92.6	35	97.2	0.82	
Non-white	1	3.7	2	7.4	1	2.8		

AO/AO–CD, adolescence-onset Conduct Disorder; CD, Conduct Disorder; CO/CO–CD, childhood-onset Conduct Disorder; CON, control; IQ, intelligence quotient; YPI, Youth Psychopathic traits Inventory. *ACORN is a geodemographic tool that assesses socioeconomic status using UK postcodes.

Participants were assessed for CD, ODD, ADHD, Major Depressive Disorder, Generalized Anxiety Disorder, Obsessive Compulsive Disorder, Post-Traumatic Stress Disorder and Substance Dependence using the Kiddie-Schedule for Affective Disorders and Schizophrenia for School-age Children-Present and Lifetime Version (K-SADS-PL; Kaufman *et al.*, 1997). Separate diagnostic interviews were performed with the participants and their caregivers and research diagnoses were made by combining information from both informants. Participants were classified as having CO–CD if they or their caregivers reported that at least one CD symptom and functional impairment was present prior to age 10 (American Psychiatric Association, 1994). If no symptoms were reported by either informant within the first 10 years of life, but they developed CD in adolescence, an AO–CD diagnosis was given. According to these criteria, 24 participants had CO–CD and 22 had AO–CD. Callous-unemotional and overall psychopathic traits were assessed using the Callous-Unemotional dimension subscale and the total score on the Youth Psychopathic traits Inventory (YPI), respectively (Andershed *et al.*, 2002; see Supplementary Material for further information and example items from this measure). Current and lifetime ADHD symptoms were assessed using the K-SADS-PL.

### fMRI task

Participants were asked to categorize the gender of grayscale photographs of angry, sad and neutral faces (half female) posed by 30 different identities (Supplementary Figure S1). The faces were selected from two stimulus sets (Lundqvist *et al.*, 1998; Tottenham *et al.*, 2009) on the basis of emotional ratings from an independent sample (Ewbank *et al.*, 2009). The stimuli were presented in 17.5 s epochs containing five faces from the same category (angry, sad or neutral) intermixed with five null events (fixation cross). Each face trial comprised a 1000-ms presentation of a face followed by a fixation cross (750 ms). Null events constituted a 1750-ms presentation of the same fixation cross. The stimuli presented during each epoch were pseudorandomized with respect to trial type (faces or null events), gender and identity; no more than three consecutive trials were of the same trial type. This pseudo-randomization enhanced design efficiency while preserving the unpredictability of stimulus onsets in naïve participants (Passamonti *et al.*, 2008). Twelve epochs of each category were presented overall (60 angry, 60 sad, 60 neutral faces; 10 min and 30 s duration in total). Reaction times (RT) and accuracy were recorded during the task.

### Image acquisition and pre-processing

MRI scanning was performed on a Siemens Tim Trio 3-Tesla magnetic resonance imaging scanner with a 16-channel head coil at the MRC Cognition and Brain Sciences Unit. Whole-brain data were acquired with echo-planar T2*-weighted imaging (EPI), sensitive to the Blood Oxygenation Level Dependent (BOLD) signal contrast (32 axial slices, 3 mm thickness; repetition time = 2000 ms; echo time = 30 ms; voxel size: 3×3×3 mm^3^). Data were analyzed using SPM5 (www.fil.ion.ucl.ac.uk/spm/). The first three volumes were discarded to allow for the effects of magnetic saturation. A high-resolution structural magnetization-prepared rapid gradient echo (MP-RAGE) scan was also acquired at a resolution of 1×1×1 mm^3^. EPIs were sinc interpolated in time to correct for slice time differences and realigned to the first scan by rigid body transformations to correct for head movements. The mean EPI was computed for each subject and inspected to ensure that no subjects showed excessive signal dropout in medial temporal and OFC regions. EPIs were coregistered and normalized to the T1 standard template in MNI space (Montreal Neurological Institute) using linear and non-linear transformations, and smoothed with a full-width-half-maximum Gaussian kernel of 8 mm.

### fMRI analyses

For each participant, a General Linear Model (GLM) assessed regionally specific effects of task parameters on BOLD indices of activation (Friston *et al.*, 1994). The model included experimental factors (angry, sad, neutral face trials and null/fixation events) and six realignment parameters as effects of no interest, to account for residual motion-related variance. Low-frequency signal drift was removed using a high-pass filter (cut-off 128 s) and an autoregressive [AR(1)] correction for serial correlations was applied.

Contrast images for the comparison angry *vs* neutral face trials were generated and entered into a second-level GLM ANOVA to produce a SPM-F map that investigated main effects of condition and group (CO–CD, AO–CD, controls); a similar ANOVA tested main effects of condition and group for the sad *vs* neutral comparison.

Finally, we assessed whether individual differences in callous-unemotional traits, overall psychopathic traits, and CD symptoms (i.e. lifetime or current symptoms) were correlated with the neural response for the angry *vs* neutral and sad *vs* neutral contrasts. This was examined in each group independently and in all 71 participants together (separate regressions were run for each personality trait and clinical symptom variable). Of note, we repeated these analyses including lifetime/ever and current ADHD symptoms as separate covariates of no interest to remove any potential confounding influence of ADHD symptoms on the results.

Two approaches for thresholding second level maps were applied. First, for a priori regions of interest (ROIs), the threshold used was *P* < 0.05, Family-Wise Error (FWE) correction for multiple comparisons in small volumes (i.e. small volume correction, svc) (Worsley *et al.*, 1996; Friston, 1997). The amygdala ROI was defined using the Talairach Daemon atlas within the Wake Forest University (WFU) PickAtlas (Lancaster *et al.*, 2000; Maldjian *et al.*, 2003). The vACC ROI was defined as a 10-mm sphere, using as a center the local maxima derived from our previous study of HCs (*x* = −8, *y* = 44, *z* = 4; Passamonti *et al.*, 2008). Brain regions that were not predicted a priori but which met a threshold of *P* < 0.001 uncorrected, *k*≥10 are reported in Supplementary Tables S1–6 for completeness.

#### PPI analysis

Variation in physiological connectivity as a function of psychological context constitutes a PPI (Friston *et al.*, 1997). PPI analysis can be used to identify regions that have differential connectivity with the source region (amygdala) according to the context (e.g. angry *vs* neutral or sad *vs* neutral faces). This was achieved using a moderator variable, derived from the product of source activation and context. In this way, regions are identified not because they are correlated with amygdala activation or the presence/absence of angry or sad faces, but because of the *interaction* between these factors. An increased amygdala response to angry *vs* neutral faces was found in both hemispheres (see Results); therefore, both left and right amygdalae were used as source regions. For each participant, a 6-mm sphere was constructed around the voxel corresponding to the anatomical center of the amygdala (left: *x* =−24, *y* = −2, *z* = −18; right: *x* = 28, *y *= −1, *z* = −19), and the time-series for each participant was computed using the first eigenvariate from all voxels’ time series in this sphere.

The BOLD time series for each participant was deconvolved to estimate a ‘neuronal time series’ for the amygdala region (Gitelman *et al.*, 2003). The PPI term (PPI regressor) was calculated as the element-by-element product of the amygdala neuronal time series and a vector coding for the main effect of task (1 for angry/sad faces, −1 for neutral faces and 0 for null events). This product was re-convolved by the canonical hemodynamic response function (hrf). The model also included the main effects of condition convolved by the hrf, and movement regressors were included as covariates of no interest. For each participant, PPI models were run and contrast images generated for positive and negative PPIs (i.e. regions showing positive or negative changes in connectivity with the source region according to context). Contrast images for each participant were then entered into a second level ANOVA with Condition (Anger > Neutral) as a within-subjects factor and Group (Controls, CO–CD, AO–CD) as a between-subjects factor. To identify the direction of the change in amygdala–vACC connectivity (i.e. negative or positive), a one-sample *t*-test was run across the whole sample. As well as testing for condition and group effects on amygdala–vACC effective connectivity, using a threshold of *P* < 0.05, FWE svc in line with previous work, we also investigated for condition and group effects on connectivity between the amygdala and the rest of the brain, using a whole-brain approach. In the latter case, we applied a voxel-wise threshold of *P* < 0.05, FWE whole-brain correction. Separate regression analyses were used to identify regions in which the changes in connectivity with the amygdala were correlated with psychopathic or CU traits, and current or lifetime CD symptoms. In all cases, a threshold of *P* < 0.05, FWE svc was used within the vACC, whereas *P* < 0.05, FWE whole-brain correction was used for areas outside the vACC. The same procedure was performed for the sad *vs* neutral contrast.

## Results

### Demographic and clinical characteristics

Table 1 provides information about the sample’s demographic and clinical characteristics. The groups were matched in age, IQ, and ethnicity, but differed in socioeconomic status, with CO–CD participants being more likely to come from lower socioeconomic status backgrounds than controls (*P* = 0.02). As expected, both of the CD subgroups scored higher than controls in overall psychopathic and CU traits, and current and lifetime CD symptoms. Post-hoc tests revealed that youths with CO–CD endorsed more lifetime CD symptoms than AO–CD youths (*P* = 0.03), but there were no other differences between the CD subgroups.

### fMRI comparisons for angry *vs* neutral faces

#### ANOVA—main effects of condition and group (HC, CO–CD, AO–CD)

Across all subjects, the main effect of condition (Angry > Neutral) was significant in both left [*F*(1, 68) = 29.91, *P* < 0.001, svc, cluster size (*k*) = 175] and right amygdala [*F*(1, 68) = 19.01, *P* < 0.005, svc, *k* = 89]. There was a main effect of group in the right [*F*(2, 68) = 6.74, *P* < 0.05, svc, *k* = 108; Supplementary Table S1), but not the left amygdala (*P* = 0.34, svc). Across the whole brain, no regions showed group differences at a threshold of *P* < 0.05, FWE whole-brain correction.

**Fig. 1.**
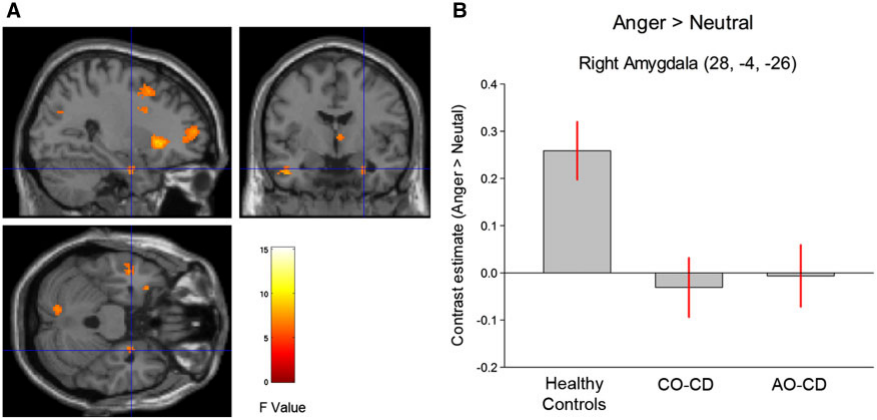
(A) Statistical parametric maps displaying the main effect of group for the contrast of angry *vs* neutral faces. Maps are thresholded at *P* < 0.005, uncorrected for display purposes only. The color bar shows *F* values. (B) Bar graphs display mean (± SE) signal change in right amygdala (Anger > Neutral) for all three groups. AO–CD indicates adolescence-onset Conduct Disorder; CO–CD, childhood–onset Conduct Disorder. Both of the Conduct Disorder subgroups showed significantly reduced right amygdala activity relative to healthy controls.

Group comparisons using *t*-tests revealed that CD subjects (collapsing across subgroups) showed a significantly lower response to Angry > Neutral compared to controls in the right amygdala [*t*(68) = 3.65, *P* < 0.01, svc, *k* = 19; Figure 1]. Considered separately, both the CO–CD and AO–CD subgroups showed a lower right amygdala response to Angry > Neutral relative to HCs [*t*(68) =−3.38, *P* < 0.02, svc, *k* = 93 and *t*(68) = −3.19, *P* < 0.03, svc, *k* = 38, respectively]. However, the CO–CD and AO–CD groups did not differ from each other in right amygdala activation (all *P*s > 0.24).

##### Influence of ADHD symptoms, and psychopathic and CU traits

An additional regression analysis, covarying out ADHD symptoms, revealed there was still a main effect of group in the right amygdala [*F*(2, 62) = 9.47, *P* < 0.01, svc, *k* = 97; n.b. ADHD symptom data were unavailable for six participants]. Again, relative to controls, participants with CD showed lower responses to Angry > Neutral in right amygdala [*t*(62) = 4.33, *P* < 0.005, svc, *k* = 210), and each of the CD subgroups showed lower right amygdala activity compared with the control group (*P*s < 0.05, svc). A further regression analysis revealed that after covarying out psychopathic traits, the group effect in the right amygdala was no longer significant (*P *>* *0.33).

### Correlations between clinical symptoms, personality measures and amygdala responses

#### CD subjects only

Current CD symptoms were positively correlated with right [*t*(44) = 2.67, *P* < 0.05, svc, *k* = 40], but not left amygdala activity (*P* = 0.09, svc). There was also a trend-level positive correlation between lifetime CD symptoms and right [*t*(44) = 2.52, *P* = 0.08, svc], but not left amygdala activity (*P* = 0.16, svc). There were no significant correlations between overall psychopathic traits or CU traits and amygdala responses in either hemisphere (all *P*s > 0.20, svc). Separate correlations in each of the CD subgroups are reported in Supplementary Material—in brief, only current CD symptoms were significantly related to amygdala activity.

### fMRI comparisons for sad *vs* neutral faces

#### ANOVA—main effects of condition and group (HC, CO–CD, AO–CD)

**Fig. 2.**
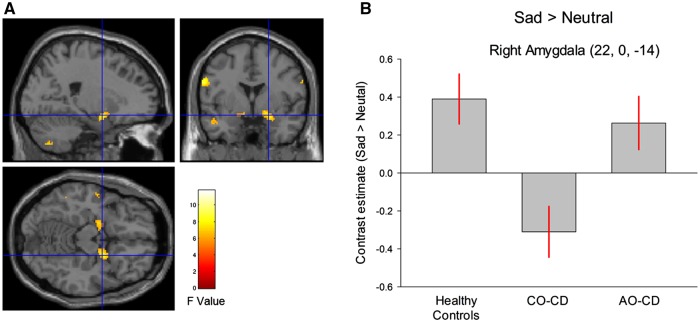
(A) Statistical parametric maps displaying the main effect of group for the contrast of sad *vs* neutral faces. Maps are thresholded at *P* < 0.005, uncorrected for display purposes only. The color bar shows *F* values. (B) The bar graphs display mean (±SE) signal change in right amygdala (Sad > Neutral) for all three groups. Key: AO–CD, adolescence–onset conduct disorder; CO–CD, childhood–onset CD; HC, healthy controls. The childhood-onset Conduct Disorder group showed significantly lower right amygdala activity to sad *vs* neutral faces relative to the other groups.

Across all subjects, the main effect of condition (Sad > Neutral) was of trend-level significance in left amygdala [*F*(1, 68) = 7.96, *P* = 0.08, svc], but not significant in right amygdala (*P* = 0.31, svc). There was a main effect of group in both the left [F(2, 68) = 7.25, *P* < 0.03, svc, *k* = 50] and right amygdala [*F*(2, 68) = 7.79, *P* < 0.02, svc, *k* = 49; Figure 2 and Supplementary Table S2).

Group comparisons using *t* tests revealed that, compared to HCs, the combined CD group showed lower responses to Sad > Neutral in right [*t*(68) = 2.88, *P* < 0.04, svc, *k* = 70] and left amygdala [*t*(68) = 3.11, *P* = 0.03, svc, *k* = 53]. CO–CD participants showed a reduced bilateral amygdala response to Sad> Neutral compared to HCs [right: *t*(68) = 3.73, *P* < 0.005, svc, *k* = 219; left: *t*(68) = 3.72, *P* < 0.005, svc, *k* = 254] and AO–CD subjects [right: *t*(68) = 3.23, *P* < 0.02, svc, *k* = 24; left: *t*(68) = 3.01, *P* < 0.03, svc, *k* = 12). There were no significant differences between controls and AO–CD subjects for this contrast (*P*s > 0.23, svc).

##### Influence of ADHD symptoms, and psychopathic and CU traits

After covarying out ADHD symptoms, there were still main effects of group in right [*F*(2, 62) = 8.04, *P* < 0.02, svc, *k* = 98], and left amygdala [*F*(2, 62) = 6.95, *P* < 0.04, svc, *k* = 91]. An independent-samples *t* test revealed that the combined CD group showed lower responses to Sad > Neutral in right [*t*(62) = 2.97, *P* < 0.03, svc, *k* = 127] and left amygdala [*t*(62) = 3.05, *P* < 0.02, svc, *k* = 150] relative to controls. CO–CD participants showed lower amygdala responses to Sad > Neutral compared to HCs [right: *t*(62) = 3.67, *P* < 0.005, svc, *k* = 205; left: *t*(62) = 3.41, *P* < 0.01, svc, *k* = 272) and AO–CD subjects [right: *t*(62) = 2.73, *P* < 0.05, svc, *k* = 50; left: *t*(62) = 2.60, *P* = 0.05, svc, *k* = 46). Again, there were no significant differences between HCs and AO–CD participants (*P*s > 0.10, svc).

All results described above remained significant when controlling for psychopathic or CU traits, except for the difference between the controls and the combined CD group in the right amygdala, which was reduced to trend-level significance when covarying out CU traits (*P* = 0.08, svc).

### Correlations between clinical symptoms, personality measures and amygdala responses

#### CD subjects only

There were no significant correlations between current or lifetime CD symptoms and right or left amygdala responses to Sad > Neutral (*P*s > 0.20, svc). There were also no significant correlations between psychopathic or CU traits and amygdala responses for this contrast (*P*s > 0.14, svc).

### Connectivity between the amygdala and ventral anterior cingulate cortex (vACC)—effects of condition and group for angry *vs* neutral faces

**Fig. 3.**
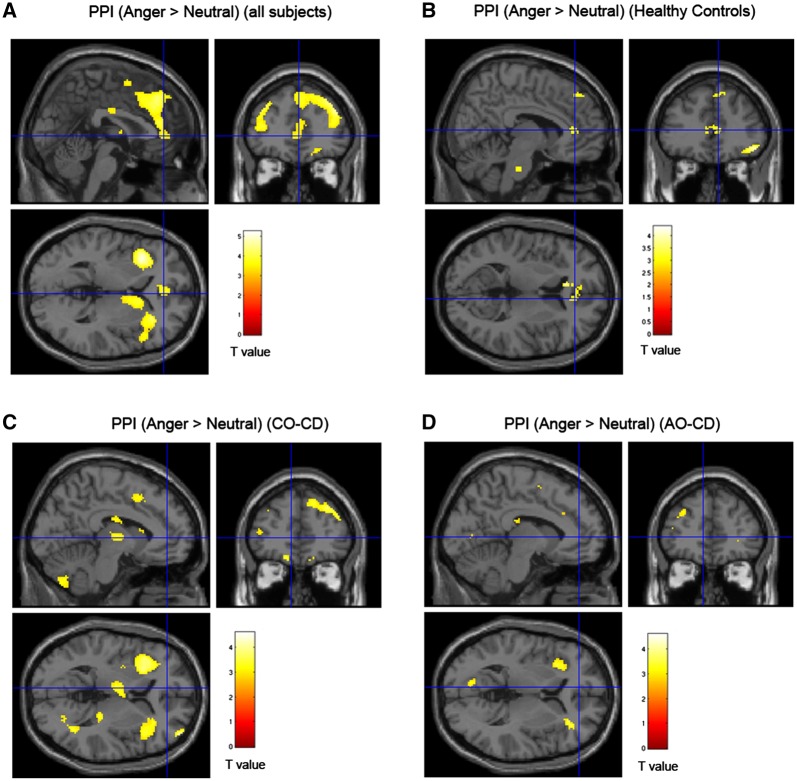
Statistical parametric maps showing changes in effective connectivity in response to angry *vs* neutral faces across all subjects and in each group separately. (A) Significant negative change in effective connectivity between the left amygdala and the vACC in response to angry *vs* neutral faces for all 71 subjects (collapsing across the HC and CD groups). (B) Regions showing a negative change in connectivity with left amygdala for HCs, (C) CO–CD subjects and (D) AO–CD subjects, respectively. Key: AO–CD, adolescence–onset Conduct Disorder; CO–CD, childhood-onset Conduct Disorder; PPI, psychophysiological interaction. Brain maps are thresholded at *P* < 0.005, uncorrected, for display purposes only and the color bars show *t* values.

Across all 71 subjects, irrespective of group, a one-sample *t*-test revealed a negative change in connectivity between vACC and left amygdala [*t*(70) = 3.72, *P* < 0.01, svc, *k* = 63; Supplementary Table S3], and also right amygdala [*t*(70) = 3.08, *P* < 0.05, svc, *k* = 77; see Figure 3A and Supplementary Table S4] during the processing of angry compared to neutral faces. There was also a marginally significant negative change in left amygdala–vACC connectivity for Anger > Neutral in the HCs alone (*P* = 0.07, svc; see Figure 3B), but not in the CD subgroups considered separately. Of interest, the negative changes in amygdala–vACC connectivity observed across all subjects were no longer significant when controlling for overall psychopathic traits (*P*s > 0.68).

An ANOVA revealed that there was no main effect of group on connectivity between vACC and either the left (*P* = 0.72, svc) or right amygdala (*P* = 0.79, svc). No regions were significant at *P* < 0.05, FWE whole-brain correction. There were still no significant main effects of group on connectivity when covarying for ADHD symptoms, psychopathic traits, or CU traits (*P*s > 0.68).

### Correlations between clinical symptoms, personality measures and amygdala–vACC connectivity

Across all 71 subjects, irrespective of group, there was no significant correlation between psychopathic traits and connectivity between vACC and left (*P* = 0.14, svc) or right amygdala (*P* = 0.18 svc). Callous–unemotional traits were also not correlated with amygdala–vACC connectivity in either hemisphere (*P*s > 0.24).

#### CD subjects only

**Fig. 4.**
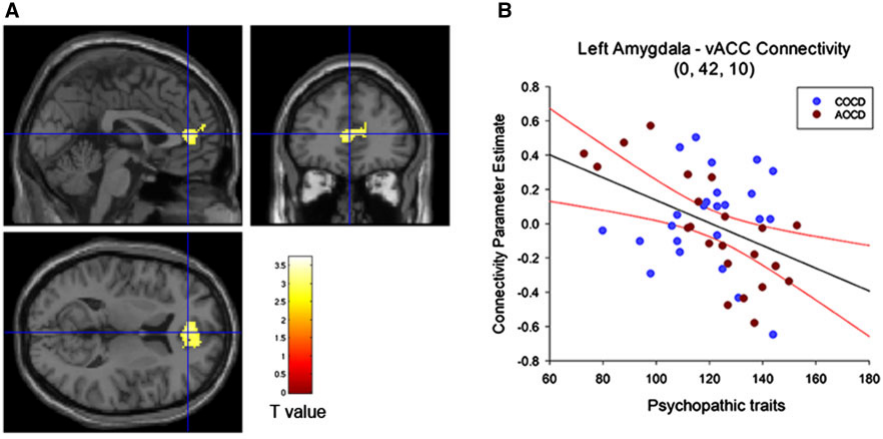
(A) Significant negative correlation between self-reported psychopathic traits and connectivity between left amygdala and vACC for angry *vs* neutral faces in the combined Conduct Disorder group (*n* = 46). The maps are thresholded at *P* < 0.005, uncorrected for display purposes only, and the color bar shows *t* values. (B) Plot shows parameter estimates of connectivity change in vACC plotted as a function of overall psychopathic traits (total YPI scores). Psychopathic traits were negatively correlated with amygdala–vACC connectivity for angry *vs* neutral faces, with participants scoring higher in psychopathic traits presenting more negative connectivity compared with lower-scoring individuals. The regression line and 95% confidence intervals are shown in black and red, respectively. AO–CD, adolescence–onset Conduct Disorder; CO–CD, childhood-onset Conduct Disorder; vACC, ventral ACC; YPI, Youth Psychopathic traits Inventory.

There was a significant negative correlation between overall psychopathic traits and left amygdala–vACC connectivity when collapsing across the CD subgroups [*t*(44) = 3.21, *P* < 0.04, svc, *k* = 235; Figure 4]. Although in the same direction, this correlation did not reach significance when using the right amygdala as a seed (*P* = 0.12, svc).

When considering the CO–CD and AO–CD groups separately, we found that psychopathic traits were significantly correlated with left amygdala–vACC connectivity in the AO–CD group alone (*P* = 0.002, svc, *k* = 470; Figure 4B). As psychopathic traits increased, the change in connectivity between the amygdala and vACC when processing angry faces became more negative (i.e. closer to the average change observed in HCs). This correlation was not significant in the CO–CD group alone (*P* = 0.29, svc), possibly because the range of YPI scores was more restricted in this subgroup (Figure 4B). In addition, there was no significant correlation between CU traits and connectivity between vACC and either left (*P* = 0.25, svc) or right amygdala (*P* = 0.29, svc).

When covarying out ADHD symptoms, the correlation between psychopathic traits and left amygdala–vACC connectivity in the combined CD group remained significant [*t*(39) = 3.16, *P* < 0.05, svc, *k* = 203], and the correlation for the right amygdala became significant [*t*(39) = 3.03, *P* = 0.05, svc, *k* = 55]. The correlation between psychopathic traits and left amygdala–vACC connectivity in the AO–CD group also remained significant [*t*(17) = 4.88, *P* < 0.01, svc], whereas the correlation in the CO–CD group was still not significant (*P* = 0.47).

The correlation between psychopathic traits and left amygdala–vACC connectivity also remained significant after factoring out current or lifetime CD symptoms (both *P* < 0.05, svc). This suggests that the effects of psychopathic traits were independent of CD symptoms, even though current CD symptoms and psychopathic traits were positively correlated (*r* = 0.37, *P* < 0.05). Lastly, there were no significant correlations between current or lifetime CD symptoms and amygdala-vACC connectivity in either hemisphere (all *P*s > 0.38).

### Connectivity between the amygdala and vACC—effects of condition and group for sad *vs* neutral faces

Across all subjects, a one sample *t*-test revealed a negative change in connectivity between vACC and left amygdala [*t*(70) = 3.12, *P* < 0.04, svc, *k* = 117; Supplementary Table S5], but not right amygdala (*P* = 0.16, svc; Supplementary Table S6). These changes in connectivity were not significant when considering the HCs alone (*Ps *>* *0.36, svc).

An ANOVA revealed that there was no main effect of group on either left or right amygdala connectivity with the vACC (*P*s > 0.91, svc), and no regions showed significant differences in amygdala connectivity between groups at *P* < 0.05, FWE whole-brain correction. No main effects of group on amygdala–vACC connectivity emerged when covarying out ADHD symptoms, psychopathic traits, or CU traits (*P*s > 0.51, svc).

### Correlations between clinical symptoms, personality measures and amygdala–vACC connectivity

There were no significant correlations between left or right amygdala–vACC connectivity and either psychopathic traits, CU traits, ADHD symptoms or current or lifetime CD symptoms (*P*s > 0.14, svc). This was the case when considering either the total sample (*N* = 71 or *n* = 65 for correlations with ADHD symptoms), or the CD group alone.

## Discussion

The aims of this study were to investigate changes in effective connectivity between the amygdala and the rest of the brain (with a particular focus on the vACC) during facial emotion processing in youths with CD, and to evaluate the impact of overall psychopathic traits, CU traits and CD symptom severity on effective connectivity. We also tested for group differences in neural responses to angry or sad *vs* neutral faces, and the effects of psychopathic traits, CU traits and CD symptom severity on neural activity. Contrary to our first hypothesis, we found that adolescents with CD did not differ from controls in terms of effective connectivity from the amygdala seed region to the vACC or the rest of the brain, and this was true when considering either angry *vs* neutral or sad *vs* neutral faces. Instead, we found a negative correlation between psychopathic traits and amygdala–vACC effective connectivity for angry *vs* neutral faces in the CD group (i.e. connectivity was *more* negative in those with higher levels of psychopathic traits). This finding was not in the direction we predicted—instead, it was the participants with CD and *lower* levels of psychopathic traits who showed the most atypical pattern of amygdala–vACC effective connectivity, whereas those with elevated psychopathic traits resembled typically developing controls (they also showed a *negative* change in connectivity when processing angry *vs* neutral faces). This relationship between psychopathic traits and effective connectivity was observed in the CD group as a whole (regardless of age-of-onset), but was particularly marked in the adolescence–onset CD group. This may reflect the fact that this group showed greater variation in psychopathic traits relative to the CO–CD group (Figure 4B). In contrast to the findings obtained for angry faces, psychopathic (or CU) traits were not significantly correlated with amygdala–vACC connectivity for sad *vs* neutral faces. Importantly, the correlations between psychopathic traits and effective connectivity for angry *vs* neutral remained significant when covarying out either current or lifetime CD symptoms (or ADHD symptoms), whereas there were no significant correlations between CD symptoms and amygdala–vACC connectivity. These results suggest that the association between psychopathic traits and amygdala–vACC functional connectivity during the processing of angry faces is partly independent of CD or ADHD symptoms. We also found no significant relationships between CU traits and amygdala–vACC connectivity, suggesting that the effects of psychopathic traits on effective connectivity were not solely driven by the affective/interpersonal aspects of psychopathy.

Consistent with our earlier findings obtained with an overlapping sample (Passamonti *et al.*, 2010), we observed group differences in amygdala activation, with CD participants showing reduced amygdala responses to angry or sad *vs* neutral faces. We also confirmed our earlier finding that the amygdala response to angry faces was lower in both of the CD subgroups, whereas these groups did not differ from each other. However, group differences in amygdala activity did not appear to be explained by group differences in amygdala–vACC connectivity. Interestingly, we did not find any significant effects of psychopathic or CU traits on amygdala responses to angry or sad faces. These findings diverge from those of previous studies showing reduced amygdala responses to fearful facial expressions in adolescents with DBDs and psychopathic traits (Marsh *et al.*, 2008) or conduct problems and CU traits (Jones *et al.*, 2009). This may be related to the use of different emotional stimuli (fearful *vs* angry expressions) or sample characteristics.

The present results suggest that the negative changes typically seen in amygdala–vACC effective connectivity during the processing of angry *vs* neutral faces are attenuated (or even shifted in the opposite direction) in adolescents with CD and low levels of psychopathic traits. This is consistent with the idea that emotion dysregulation plays an important role in the etiology of antisocial behavior which occurs in the absence of psychopathic traits—deficits in the regulation of emotional responses, possibly due to impairments in top-down control mechanisms, are argued to lead to excessive reactions to provocation and reactive aggression (Frick and Viding, 2009). Over time, this may lead to an increased risk for disorders related to emotion dysregulation, such as anxiety or depression, amongst this group (Frick and White, 2008). It is interesting that the association between psychopathic traits and aberrant effective connectivity was specific to angry faces, which are typically considered threat cues—one possibility is that regulatory control mechanisms are more strongly engaged when processing angry faces, and therefore the difficulties found in those with CD and lower levels of psychopathic traits are more apparent under such conditions. In contrast, sad faces represent relatively unambiguous distress cues. Previous behavioral studies have shown more pronounced anger recognition deficits in youth with antisocial behavior without psychopathic traits than their psychopathic counterparts (Dadds *et al.*, 2006). This study also demonstrates that healthy adolescents show a significant (negative) change in effective connectivity between the amygdala and vACC during the processing of angry *vs* neutral faces, replicating findings in healthy adults (Passamonti *et al.*, 2008).

This study had several strengths. It included one of the largest CD samples in the fMRI literature (Alegria *et al.*, 2016; although see Aghajani *et al.*, 2017). The participants were also well-characterized in terms of comorbid disorders, psychopathic traits and CU traits and the CD and control groups were matched in age and IQ. We note that the study was restricted to male participants, which limits the generalizability of the findings, but also ensures the results are easier to interpret, given that sex differences in brain activity during face processing have been reported (Fusar-Poli *et al.*, 2009; Schneider *et al.*, 2011). In terms of limitations, the cross-sectional design means that we cannot infer a causal relationship between (low) psychopathic traits and changes in amygdala–vACC connectivity. We note that our CD group was a selected sample of young people who had either been arrested or excluded from school, thus it is possible that a weaker relationship between psychopathic traits and effective connectivity would be observed in a more representative sample. We also performed a large number of comparisons between groups and across multiple behavioural and personality measures, correcting the results at the single analysis level. Nevertheless, we had specific hypotheses regarding amygdala–vACC connectivity, and many of the subsidiary analyses were performed to test the impact of potential confounding variables, such as ADHD comorbidity. Finally, although the YPI is a widely-used measure of juvenile psychopathic traits, relying solely on self-report measures of psychopathic traits is not optimal as young people with CD may lie or lack insight into their own personality characteristics. Future studies should therefore collect multiple measures of psychopathic traits, and use both self-report and parent-report questionnaires.

## Conclusions

We found that changes in effective connectivity between the amygdala and vACC during the processing of angry faces were negatively correlated with psychopathic traits, such that functional coupling between these regions was less negative in the CD participants with lower levels of psychopathic traits than in those with elevated psychopathic traits. However, there were no significant correlations between psychopathic or CU traits and amygdala activation to angry (or sad) faces. In contrast, participants with CD (regardless of age-of-onset) showed reduced bilateral amygdala responses to angry *vs* neutral faces, but did not differ from controls in effective connectivity between the amygdala and associated brain regions. These results provide some of the most convincing evidence available to date that the effects of CD and psychopathic traits on neural activity and functional interactions between brain regions are at least partly dissociable. These findings also emphasize the importance of using effective connectivity methods to characterize the neural mechanisms underlying CD and psychopathic traits.

## Supplementary data

Supplementary data are available at *SCAN* online.

## Funding

This study was supported by the Medical Research Council (MRC) through project code MC-A060-5PQ50 (to A.J.C.), a project grant from the Wellcome Trust (083140 to G.F. and I.M.G.), the Betty Behrens Research Fellowship at Clare Hall, University of Cambridge (to L.P.) and MRC research grant MR/P01271X/1 (to L.P.). 

*Conflict of interest*. None declared.

## Supplementary Material

Supplementary Data
