# Supplementary material for: Psychopathic traits influence amygdala–anterior cingulate cortex connectivity during facial emotion processing
Source: Soc Cogn Affect Neurosci. 2018 Apr 12;13(5):525–34. doi: 10.1093/scan/nsy019 (PMC6007413; doi:10.1093/scan/nsy019)
Supplement: Supplementary Data [file nsy019_scan-17-334.pdf]

		MNI Coordinates				
**Cerebral Region**	**Side**	**X**	**Y**	**Z**	**Cluster size**	**F-value**
Amygdala	R	28	-4	-26	108	6.74**
Dorsolateral prefrontal cortex	R	50	28	34	1052	15.18*
Dorsal anterior cingulate cortex	R	10	26	44	407	12.59*
Inferior parietal lobe	R	48	-48	46	96	10.94*
Claustrum	R	28	20	2	30	9.22*
Thalamus	R	4	-10	4	14	8.70*

Supplementary Table 1. Regions showing a main effect of group for the contrast anger > neutral. Significant at *p<0.001, uncorrected, *k*≥10, **p<0.05, Family-Wise Error, small volume correction. MNI, Montreal Neurological Institute.

		MNI Coordinates				
**Region**	**Hemi-sphere**	**X**	**Y**	**Z**	**Cluster size**	**F-value**
Amygdala	R	22	0	-14	49	7.79**
	L	-18	-6	-12	50	7.25**
Inferior temporal cortex	L	-60	-14	-24	56	11.76*
	R	56	-10	-22	11	8.47*
Precentral gyrus	L	-60	4	30	11	11.17*
Posterior insula	L	-44	-18	22	23	9.84*

Supplementary Table 2. Regions showing a main effect of group for the contrast sad > neutral. Significant at *p<0.001 uncorrected, *k*≥10; **p<0.05, Family-Wise Error, small volume correction. MNI, Montreal Neurological Institute.

		MNI Coordinates				
**Cerebral Region**	**Side**	**X**	**Y**	**Z**	**Cluster size**	**T-value**
Ventral anterior cingulate cortex		0	42	2	63	3.72**
Anterior insula	L	-34	18	4	1429	4.93*
	R	42	22	-4	1275	4.44*
Dorsolateral prefrontal cortex	R	32	50	34	3572	4.50*
Anterior temporal lobe	R	36	8	-38	457	4.43*
Inferior temporal lobe	R	60	-30	-20	25	3.70*
Caudate	R	12	16	-20	16	3.42*

Supplementary Table 3. Regions showing a negative change in connectivity with the left amygdala for the contrast anger > neutral across all 71 subjects. Significant at *p<0.001, uncorrected, *k*≥10, **p<0.05, Family-Wise Error, small volume correction. MNI, Montreal Neurological Institute.

		MNI Coordinates				
**Cerebral Region**	**Side**	**X**	**Y**	**Z**	**Cluster size**	**T-value**
Ventral anterior cingulate cortex		0	42	0	77	3.08**
Posterior cingulate cortex	R	14	-30	30	1013	5.38*
Dorsal anterior cingulate cortex	L	-10	20	36	1130	4.54*
Posterior mid cingulate cortex	L	-8	-20	30	96	4.10*
Dorsolateral prefrontal cortex	R	34	50	32	62	3.93*
	L	-34	42	36	86	3.86*
Ventrolateral prefrontal cortex	R	40	52	-10	46	3.78*
Dorsal posterior insula	L	-38	-14	22	60	3.73*
Anterior superior temporal gyrus	R	54	4	-10	39	3.72*
Right middle frontal gyrus	R	34	8	40	89	3.71*
Ventromedial prefrontal cortex	R	26	22	-20	49	3.68*
	L	-20	28	-16	11	3.34*
Caudate	R	10	14	0	75	3.68*
Anterior insula	L	-42	18	4	131	3.64*
Precentral gyrus	L	-56	2	22	33	3.56*
Inferior temporal cortex	R	60	-22	-24	14	3.50*
Paracentral lobule	L	-16	-38	54	11	3.48*
Ventromedial prefrontal cortex	L	-20	28	-16	11	3.34*

Supplementary Table 4. Regions showing a negative change in connectivity with the right amygdala for the contrast anger > neutral across all 71 subjects. Significant at *p<0.001, uncorrected, *k*≥10, **p<0.05, Family-Wise Error, small volume correction. MNI, Montreal Neurological Institute.

		MNI Coordinates				
**Region**	**Hemi-sphere**	**X**	**Y**	**Z**	**Cluster size**	**T-value**
Ventral anterior cingulate cortex	L	-4	40	2	117	3.12**
Occipital cortex	R	48	-80	14	137	4.65*
Precentral gyrus	L	-28	0	38	115	4.04*
Caudate	L	-14	28	4	258	3.91*
Orbitofrontal cortex	L	-18	24	-18	27	3.87*
	R	24	20	-18	13	3.63*
Ventromedial prefrontal cortex	L	-14	50	-12	37	3.82*
	R	22	38	-10	12	3.56*
Cerebellum	L	-30	-44	-28	52	3.77*
Dorsolateral prefrontal cortex	R	36	60	14	141	3.76*
Inferior parietal cortex	R	52	-30	36	155	3.68*
Middle frontal gyrus	R	36	38	40	40	3.63*

Supplementary Table 5. Regions showing a negative change in connectivity with the left amygdala for the contrast sad > neutral. Significant at *p<0.001 uncorrected, *k*≥10; **p<0.05, Family-Wise Error, small volume correction. MNI, Montreal Neurological Institute.

		MNI Coordinates				
**Region**	**Hemi-sphere**	**X**	**Y**	**Z**	**Cluster size**	**T-value**
Middle temporal gyrus	R	42	-6	-24	15	3.48*

Supplementary Table 6. Region showing a negative change in connectivity with the right amygdala for the contrast sad > neutral. Significant at *p<0.001 uncorrected, *k*≥10. MNI, Montreal Neurological Institute.

Supplementary Information for Ewbank et al. ***Psychopathic traits influence amygdala-anterior cingulate cortex connectivity during facial emotion processing*****.**

Questionnaires

Psychopathic and callous-unemotional traits were assessed using the total score and the CU dimension subscale of the self-report Youth Psychopathic traits Inventory (YPI; Andershed et al., 2002), respectively. The YPI contains 50 items assessing manipulative, grandiose, and unemotional traits and behaviors, each answered on a four point scale from 'Does not apply at all' to 'Applies very well'. Example items include: 'I seldom regret things I do, even if other people feel that they are wrong', 'I often become sad or moved by watching sad things on TV or film' (reverse scored) and 'I don't let my feelings affect me as much as other people's feelings seem to affect them.' Total scores on the YPI range from 50-200, with higher scores indicating higher levels of psychopathic traits. The Cronbach's alphas for the YPI total score and CU dimension subscale were 0.88 and 0.74, respectively (Andershed et al., 2002).

Correlations between clinical symptoms, personality measures and amygdala responses to Angry versus Neutral in the Conduct Disorder (CD) subgroups considered separately

Current CD symptoms

In the childhood-onset CD (CO-CD) group, there was a significant correlation between activity and current CD symptoms in right amygdala ($t(22)=3.02$, $p < 0.05$ svc, k=19) and left amygdala ($t(22)=3.02$, $p < 0.05$ svc, k=13). There were no significant correlations between amygdala activity and current symptoms in the adolescence-onset (AO-CD) group considered alone ($ps > 0.48$).

Lifetime CD symptoms

The CO-CD group showed no significant correlation between right amygdala ($p=0.16$) or left amygdala ($p=0.41$) and lifetime CD symptoms. There were no significant correlations between amygdala activity and lifetime symptoms in the AO-CD group ($ps >0.56$).

Psychopathic traits (total YPI)

The CO-CD group showed no significant correlation between psychopathic traits and right ($p=.36$) or left amygdala activity ($p=.18$). There were no significant correlations between right ($p=.15$) or left amygdala ($p=.48$) activity and psychopathic traits in the AO-CD group.

CU traits

The CO-CD group showed no correlation between CU traits and right ($p=.45$) or left amygdala ($p=.33$) activity. Likewise, CU traits were not correlated with right ($p=.23$) or left amygdala ($p=.36$) activity in the AO-CD group considered separately.

References

Andershed, H., Kerr, M., Stattin, H. & Levander, S. (2002). Psychopathic traits in non-referred youths: A new assessment tool. In: Blaauw E., & Sheridan, L., (Eds.). *Psychopaths: Current International Perspectives.* Hague, Netherlands: Elsevier.
